# Post-Concussive Symptoms and Their Association with Follow-Up Care in a Sample of Young Children with TBI

**DOI:** 10.3390/children13091150

**Published:** 2026-08-27

**Authors:** Jennifer Coto, Ivette Cejas, Nicholas Smith, Alina Farias, Juan Pablo Solano, Dainelys Garcia

**Affiliations:** 1 Department of Pediatrics, University of Miami Miller School of Medicine, Miami, FL 33136, USA; icejas@med.miami.edu (I.C.);; 2 Department of Otolaryngology, University of Miami Miller School of Medicine, Miami, FL 33136, USA; 3 Department of Psychiatry and Behavioral Neurosciences, University of South Florida Health Morsani College of Medicine, Tampa, FL 33613, USA; smithn1@usf.edu

**Keywords:** concussion, pediatrics, post-concussive symptoms, follow-up, traumatic brain injury

## Abstract

**Highlights:**

**What are the main findings?**

- Fewer than half of families sought follow-up care after their child’s emergency department visit for TBI, and only one-third of those who followed up were seen by a TBI specialist.
- Among individual post-concussive symptoms, balance difficulties and drowsiness remained significantly associated with seeking follow-up care.

**What are the implications of the main findings?**

- Young children with TBI may not receive appropriate post-injury follow-up, particularly specialty care, even when post-concussive symptoms are present.
- Caregiver education and first-line provider training are needed to improve recognition of post-concussive symptoms, especially less observable symptoms, and to strengthen referral pathways to TBI specialty care.

**Abstract:**

Background: Pediatric traumatic brain injury (TBI) is the leading cause of acquired disability in childhood and is associated with post-concussive symptoms (PCS) that may negatively impact recovery and long-term outcomes. Despite the importance of follow-up care after TBI, families from marginalized racial and ethnic backgrounds are less likely to receive follow-up care and rehabilitation. This study examined the association between PCS and follow-up care, as well as the types of follow-up care received, among young, racially and ethnically diverse children with TBI. Methods: Data were collected from 36 families of children ages 2–5 years with a confirmed TBI in the past 3–6 months who completed screening procedures as part of a larger study. Parents reported demographic and injury characteristics, PCS, and whether they sought follow-up care after their child’s emergency department visit. Regression analyses examined associations between PCS and follow-up care. Results: Participants were 55.6% female, with a mean age of 3.60 years (*SD* = 1.22). Most children sustained mild injuries due to falls (77.8%). Headaches, drowsiness, balance difficulties, feeling slowed down, and behavior changes were individually associated with seeking follow-up care. In a regression model including all significant main effects, balance difficulties (*B* = 0.46) and drowsiness (*B* = 0.42) remained significant [*F*(5,30) = 6.11, *p* < 0.001]. Only 44.4% of parents sought follow-up care, and 33.3% of them followed up with a TBI specialist. Conclusions: Findings highlight low rates of post-injury follow-up and specialty care among young children with TBI, underscoring the need for improved caregiver education and first-line provider training.

## 1. Introduction

Traumatic brain injury (TBI) is the leading cause of acquired disability in childhood, affecting over 511,000 children between the ages of 0 and 14 years annually [1]. Young children experience the highest rates of TBI-related emergency department visits [2], with mild TBI (mTBI), representing approximately 70–90% of all TBIs [3,4]. Although young children’s brains may be better able to adapt to some effects of head injuries because of greater neuroplasticity, research suggests that children injured in early childhood (<7 years) may also experience significant long-term consequences (e.g., behavior problems, executive dysfunction, and learning problems) [5]. To date, pediatric TBI research has primarily focused on understanding of physiological responses and patterns of recovery for children with TBI to aid in development of treatment guidelines. However, despite the recent increase in attention directed towards pediatric TBI, research is needed to help professionals better predict the acute and long-term needs of children following concussion/mild TBI in order for providers to appropriately support families through the course of recovery.

Research examining predictors of long-term outcomes for children who have sustained an mTBI suggests that the onset, persistence, and severity of post-concussive symptoms (PCS) has been linked to post-injury recovery and long-term outcomes [6]. PCS may include cognitive (e.g., difficulty concentrating, difficulty remembering), behavioral (e.g., irritability), emotional (e.g., sadness, nervous), or physical symptoms (e.g., nausea/vomiting, headaches, dizziness, drowsiness, and sensitivity to noise or light) that have been associated with complex long-term effects on the course of injury recovery and quality of life for the child and family [6,7,8,9]. Specifically, PCS have been associated with a decline in cognition and academic performance, and difficulties with behavior, socialization, and adaptive functioning [10]. While PCS often resolve within several weeks following an injury, for a subset of children (as high as 31%), PCS can persist for months and sometimes years following the injury [11].

Timely and appropriate follow-up care is a critical part of post-injury recovery and has been highlighted as an important avenue for mitigating PCS and negative long-term outcomes [12]. Initial care following mTBI typically occurs in the emergency department [13], and while outpatient follow-up after (e.g., specialty care clinic) is clinically recommended for ongoing assessment and management of PCS for children with mTBI [14], less is known about the relation between PCS and rates of follow-up care. Despite the documented negative impacts that the lack of appropriate and timely TBI management may have on unmet/unrecognized needs [12], few studies to date have examined the association between PCS and follow-up care, particularly during the post-injury recovery period. To date, one study found a non-significant tendency toward increased follow-up care among children with persistent PCS when compared to those without persistent PCS. Although notably, less than half of the sample attended follow-up visits, and those with persistent PCS had a greater number of missed school days [15]. These findings highlight concerning rates related to follow-up care and suggests children with PCS may benefit from additional resources and support that are more likely to be received when engaging in follow-up care.

In addition, research has shown significant disparities in disability and functional outcomes between Hispanic and non-Hispanic children. Specifically, Hispanic children are more likely to sustain more severe injuries, be younger at the time of injury, and report more long-term reductions in quality of life and adaptive skills, even when adjusting for injury severity, insurance, parent education, and income [16]. Despite the greater risk for negative long-term outcomes (e.g., behavior problems, executive dysfunction, and learning problems) [4,17], families from marginalized racial and ethnic backgrounds, particularly Hispanic and Black families, have been shown to be less likely to receive follow-up care and rehabilitation following a TBI [18]. Given the negative impact PCS can have on the course of mTBI recovery and long-term academic, emotional, physical, and behavioral outcomes, and the lower rates of follow-up care among families from marginalized racial and ethnic backgrounds, research is needed. The current study addresses a major gap in the literature by examining the type of follow-up care received by families during the post-injury recovery period, as well as the association between PCS and the likelihood of seeking follow-up care among young ethnically diverse children with TBI and their families.

## 2. Methods

### 2.1. Procedures

Participants were recruited over a 6-month period as part of a larger study examining Parent–Child Interaction Therapy (PCIT) in children with TBI [19]. Recruitment took place in the Emergency Department at a pediatric hospital in the southeastern United States. Families of children with a confirmed TBI within the past 3–6 months and aged 2–5 years were contacted via telephone and screened to determine eligibility for the study. Subsequently, parents (*n* = 36) completed subscales of the Child Behavior Checklist (CBCL) and were scheduled for an in-person screening if their child’s subscale scores fell in the clinically elevated range per larger study inclusion criteria. Written consent was provided during the in-person assessments. Children were excluded if the child had physical deficits which significantly impacted their mobility or cognitive deficits that were severe enough to prevent participation in PCIT as part of the larger study. For the purposes of this study, assessments were completed by the child’s primary parent during both the telephone and in-person screening.

### 2.2. Participants

The sample consisted of 36 families of children ages 2–5 years with documented evidence of a TBI within the past 3–6 months, with at least one physical finding consistent with head trauma (i.e., loss of consciousness [LOC], remarkable computed tomography scan or magnetic resonance imaging, or physical symptoms consistent with head injury in children reported by caregivers/and or doctors, such as vomiting and drowsiness). All 36 children completed screening for the larger study and were included in the present analyses. As part of the larger study, personnel reviewed emergency department reports from a large, urban pediatric hospital selected due to its existing specialty TBI clinic. Families of children aged 2–5 years with documented evidence of a TBI within the past 3–6 months were actively recruited via a brief telephone screening that consisted of completing the Aggressive and Attention subscales of the Child Behavior Checklist (CBCL). If the primary caregiver rated the child’s behavior as elevated (i.e., T-score 60) on either subscale, the family was scheduled to complete a second screening at a university-based clinic. See [masked for review] for additional information related to the larger PCIT study. Full demographic information was available only for the subset of children who went on to enroll in the larger PCIT study and completed the baseline visit (*n* = 15). The remaining 21 children completed screening procedures but did not enroll in the larger study; therefore, full demographic data were not available for these participants. Children had a mean age of 3.60 (*SD* = 1.22) and over half were female (55.6%). Of note, demographic information for parents was limited to participants who completed the baseline visit for the larger study (*n* = 15). All parents were mothers with a mean age of 29.47 (*SD* = 5.37), most of which were never married (46.7%) and attended some college/technical school (53.3%). Mothers endorsed their ethnicity as mostly Hispanic (73.3%) and race as White (80%). See Table 1 for further descriptive information.

### 2.3. Measures

#### 2.3.1. Demographics and Injury Description

Demographic information was collected via parent report: child age, child gender, child ethnicity, child race, parent age, parent gender, parent ethnicity, parent race, marital status, and parent level of highest education. Additionally, parents provided descriptive injury data: injury date, mechanism of injury, injury severity, and indications of if the child experienced any LOC. Lastly, parents provided information on whether they sought follow-up care after their child’s emergency room visit, and if so, with what type of provider (e.g., primary care, TBI specialist).

#### 2.3.2. Post-Concussive Symptoms

The Post-Concussive Symptom Inventory (PCSI-Parent report) is a 16-item measure used to assess post-concussive symptoms following TBI. For the purposes of this study and given the age of the children in the sample, parents completed the PCSI. The PCSI yields a total score as well as four factor scores: Physical, Fatigue, Emotional, and Cognitive. The PCSI is a reliable and valid measure [20]. Higher scores are indicative of more PCS.

### 2.4. Data Analyses

All analyses were conducted using the Statistical Package for the Social Sciences (SPSS; version 26). Preliminary analyses were conducted between demographic variables and all outcome variables to identify any associations and no significant associations were found. Next, descriptive analyses were conducted for demographic and outcome variables. A regression analysis was conducted between LOC and PCS. Prior to conducting the linear regression, residual plots and influence statistics were examined. The residuals demonstrated some departure from normality, although no standardized residual exceeded ±3. Inspection of the standardized residuals plotted against predicted values did not indicate a pronounced pattern of heteroscedasticity. Cook’s distance and leverage values did not identify any highly influential observations. Additionally, a regression analysis was conducted between individual PCS and follow-up care. Lastly, a regression model including all significant main effects was conducted. Of note, given that the present study was a secondary analysis of data collected as part of a larger parent study, an a priori sample size calculation was not conducted specifically for the current analyses.

## 3. Results

### 3.1. Injury Characteristics, Post-Concussive Symptoms, and Follow-Up Care

Injury severity and mechanism varied across the sample, though the majority sustained mild injuries (83.3%) due to falls (77.8%). Over half of the sample (52.8%) underwent CT/MRI scans during their emergency room visit, with 37.5% (*n* = 6) of those children having a positive finding in their imaging. One quarter of children experienced LOC during their injury. Overall, children had a mean of 2.81 post-concussive symptoms (*SD* = 2.91). Individual symptoms endorsed were: changes in behavior (44.4%), nausea/vomiting (41.7%), headaches (38.9%), balance difficulties (25%), drowsiness (22.2%), feeling slowed down (19.4%), difficulty falling asleep (13.9%), sleeping less (11.1%), sensitivity to noise (11.1%), sadness (11.1%), dizziness (11.1%), being difficult to console/irritability (8.3%), sensitivity to light (5.6%), vision problems (5.6%), sleeping more (5.6%), and memory loss (5.6%). Furthermore, less than half of the parents sought follow-up care after their child’s emergency room visit (*n* = 16; 44.4%), and of those, only 33.3% (*n* = 5) followed up with a TBI specialist. See Table 1 for further descriptive information.

### 3.2. Loss of Consciousness and Post-Concussive Symptoms

A regression analysis was conducted to examine whether loss of consciousness significantly predicted total post-concussive symptoms. Results of the regression indicated that loss of consciousness was significantly associated with PCS, *F*(1,34) = 34.71, *p* < 0.001, *R*^2^ = 0.51, *B* = 4.7. Children who had experienced loss of consciousness had a higher number of post-concussive symptoms.

### 3.3. Post-Concussive Symptoms and Follow-Up Care

Regression analyses were also used to examine the association between PCS and seeking follow-up care with a medical professional. Headaches (*B* = 0.44), drowsiness (*B* = 0.55), balance difficulties (*B* = 0.59), feeling slowed down (*B* = 0.51), and behavior changes (*B*= 0.44) were all individually associated with seeking follow-up care.

Lastly, a regression model including all significant main effects was conducted. Difficulties with balance (*B* = 0.46) and drowsiness (*B* = 0.41) remained significant, indicating that parents were more likely to follow up if their child had difficulties with balance and drowsiness [*F*(5,30) = 6.12, *p* < 0.001]. See Table 2.

## 4. Discussion

The current study examined the association between post-concussive symptoms (PCS) and the likelihood of seeking follow-up care among young, ethnically diverse children with TBI and their families. Overall, 75% of children experienced PCS, yet less than half of families sought follow-up care after the emergency department visit. Children who experienced loss of consciousness (LOC) had a higher number of PCS, and families were more likely to seek follow-up care when their child experienced balance difficulties and drowsiness. These findings underscore the importance of caregiver recognition of symptoms and continued monitoring following concussion, particularly among young children who may have difficulty recognizing or verbally reporting their symptoms.

Although often considered a relatively minor injury, mTBI can result in serious and potentially lasting problems, underscoring the importance of appropriate monitoring and follow-up care [21]. PCS can negatively impact the course of mTBI recovery and long-term academic, emotional, physical, and behavioral outcomes. Specifically, previous research has found that more severe acute symptoms are predictive of prolonged recovery [22]. Experiencing PCS for long periods of time can have a detrimental effect on emotional well-being and can make it difficult for children to perform well in school. This is particularly concerning when children are not receiving specialized follow-up care during recovery, as follow-up providers can monitor symptoms and facilitate appropriate school accommodations. In fact, previous research has found that children with prolonged PCS missed twice as many days at school compared to those without PCS, yet were not more likely to receive school accommodations [15]. Moreover, there was an association with those who received follow-up care being more likely to receive school accommodations, underscoring the need for follow-up care after concussion [15]. Despite these known effects, less than half of the families in the current study sought follow-up care, suggesting that many young children may not receive continued monitoring during a period when symptoms can affect functioning and recovery.

Overall, 75% of children in this study reported experiencing PCS, with the most common symptoms being changes in behavior (44.4%), nausea/vomiting (41.7%), headaches (38.9%), and balance difficulties (25%). Changes in behavior reflected a single parent-reported PCSI item and did not assess the specific nature of the behavioral change. Therefore, it was not possible to determine whether caregivers observed increased tantrums, loss of interest, developmental regression, or other behavioral manifestations. The frequency of reported behavioral changes may reflect the developmental characteristics of the sample, as young children may be unable to describe internal symptoms and may instead express distress through changes in behavior or daily functioning. Moreover, children who experienced LOC had a higher number of PCS, which is consistent with the prior literature [23,24]. This finding may indicate that LOC is associated with a more clinically apparent injury presentation or greater acute symptom burden. Additionally, six children had reported positive CT/MRI findings; however, the available emergency department records did not provide sufficient detail to characterize the specific abnormalities, limiting our ability to assess their clinical significance. However, LOC did not necessarily result in families obtaining follow-up care, suggesting that the injury event alone may not be sufficient to prompt continued care.

Despite LOC and increased reports of PCS, only 44.4% of families enrolled in this study sought follow-up care by any provider. These low rates are consistent with Fridman et al., 2018 who found a follow-up rate of 30.1% in children ages 3–18 years in Canada [25]. The difference between studies may reflect variation in participant age, healthcare systems, injury characteristics, emergency department discharge practices, or access to follow-up providers. Nevertheless, both studies demonstrate that a substantial proportion of children do not receive continued care following concussion. The Berlin Consensus Statement on Concussion in Sport describes a protocol consisting of a short period of physical and cognitive rest, followed by a gradual return to physical and cognitive activity [13]. Additionally, health professionals should monitor the recovery process and provide final clearance before returning to full activity. As our findings demonstrate, more than half of patients are not following up with any provider and only 33% of those who are receiving follow-up care are seeing a TBI specialist. This is concerning because providers without specialized TBI training may be less familiar with evolving concussion-management recommendations. For example, previous recommendations included longer periods of physical and cognitive rest after injury while more recent recommendations include a swifter return to physical and cognitive activity below increased symptom thresholds [13]. More importantly, providers with limited TBI experience may clear children to return to physical activity prematurely, potentially increasing the risk of symptom exacerbation, prolonged recovery, or repeat injury [26,27]. Ultimately, without proper follow-up, concussion recovery can be prolonged, and symptoms may be exacerbated.

Moreover, our study found that families were likely to follow up with a provider if their child experienced balance problems and drowsiness. These findings may reflect the observable nature of these symptoms. Balance difficulties and drowsiness may be more noticeable to caregivers than internal symptoms such as headache, dizziness, slowed thinking, or emotional changes, particularly when young children cannot clearly describe what they are experiencing. To our knowledge, this is the first study to examine the association between specific PCS and follow-up care utilization in young children. However, a study by Stevens and colleagues (2010) examining parent symptom identification after emergency department discharge found that most parents were unable to identify PCS in their children, even when they received verbal and written discharge instructions [28]. When looking at the symptoms individually, headache was the most commonly observed (37%) while balance was only reportedly observed by 2.7% of parents [28]. The higher frequency of balance difficulties in the present study may reflect differences in sample age, symptom-assessment methods, injury characteristics, or the timing of assessment. However, both studies suggest that parents may have difficulty recognizing the full range of symptoms that can occur following concussion. These data demonstrate that there is a lack of knowledge regarding PCS and concussion even after receiving written materials. Educating parents on the importance of recognizing PCS is crucial as management of symptoms is imperative in the recovery process. This is especially important for young children as it may be difficult for children to report symptoms. Recent research by Dupont and colleagues (2024) further suggests that concussion symptoms in infants and preschool-aged children may differ from those observed in older children and may be expressed through developmentally specific changes in behavior and functioning that require caregiver observation [29]. Measures developed specifically for young children, such as the Report of Early Childhood Traumatic Injury Observations and Symptoms inventory, may therefore improve the identification of symptoms that are not adequately captured by measures designed for older children [29]. Because the current study used the PCSI, some developmentally specific behavioral or functional manifestations may have been underidentified. Notably, parents in our sample followed up with a provider due to observable symptoms (e.g., balance problems, drowsiness). However, many PCS are difficult to observe, and therefore evaluation and management by a TBI specialist is essential. Future research should focus on identifying effective ways for first-line providers to educate parents about both observable and less visible PCS and should consider incorporating symptom measures specifically designed for young children.

### 4.1. Clinical Implications and Future Directions

Our study highlights the paucity of post-injury follow-up care by TBI specialists. Given the literature on the possibility of PCS persisting and/or worsening particularly in the first year after injury, initiatives need to target increasing awareness of concussion, as well as streamlining the referral pathways for this underserved pediatric population. Emergency department discharge procedures could include standardized, developmentally appropriate education, explicit recommendations for follow-up, and direct referrals to primary care or pediatric TBI specialists when available. This is even more critical for Black/African American athletes who have been shown to return to sports or school earlier than White athletes [30]. Although there is limited research on why there is this racial difference, some studies have hypothesized that this may be driven by less concussion knowledge, insurance barriers, and negative attitudes toward concussion. As previously stated, returning to physical activity before concussion symptoms have resolved can be detrimental as it is linked with poorer overall outcomes (e.g., prolonged recovery, cognitive issues).

All children who have had a concussion should be followed and managed throughout their recovery period. Future work needs to focus on educating parents, teachers, and sports staff, as well as first-line providers who come into contact with the families at the onset of their diagnosis and treatment (i.e., emergency department providers). This is critical as first-line providers frequently diagnose concussion but lack adequate training [31]. Additionally, educating primary care providers on the complexities of concussion recovery and importance of specialized treatment so that appropriate referrals are made is warranted.

### 4.2. Limitations

While this study helps to address gaps in the pediatric mTBI literature, there are important limitations to consider. First, this sample comprised children with elevations on the CBCL aggression or attention subscales due to the inclusion criteria for the larger study, and therefore, results may not generalize to children with TBI without externalizing behaviors. However, previous research has found an association between behavior problems and children with TBI, and therefore, results may still be applicable to most children with mTBI [32]. Second, given the age of the participants, we relied on parent reports of PCS, and therefore, parents may have under- or overreported their child’s symptoms. Nonetheless, PCSI parent report is a measure commonly used in research in this age group [20]. Third, although the sample was relatively balanced by sex, with 55.6% of participants identified as female, the modest sample size precluded adequately powered comparisons of post-concussive symptoms by sex. Future studies with larger samples should examine whether sex influences symptom presentation, severity, and follow-up-care utilization. Fourth, we did not collect information on where parents were referred to after their emergency department visit, and therefore we are unable to determine whether parents were referred to their primary care physician or to a specialty clinic. Although standard protocol for the emergency department where recruitment was conducted was to refer to their hospital-affiliated TBI specialist post-emergency department visit, our data showed that this was not consistently occurring, or parents were not following these recommendations. Lastly, because the present study used screening data from a larger PCIT study, full demographic information was available only for participants who enrolled in the larger study and completed the baseline visit (*n* = 15). The remaining participants completed screening procedures but did not enroll in the larger study, limiting the availability of demographic covariates. However, all 36 participants had injury, symptom, and follow-up data and were included in the present analyses. Future research should collect complete demographic information for all screened participants to allow for more robust examination of demographic factors as covariates.

## 5. Conclusions

In conclusion, our study highlights that despite the identified public health concern, pediatric mTBI patients are not receiving the necessary specialized care. Children with concussions often experience symptoms that may range from mild to debilitating, which may resolve quickly with proper care or be prolonged. Education on the symptoms, how to identify them, and proper course of treatment is drastically needed to improve TBI management in children. In addition, TBI specialists need to work with first-line providers, the sports community, and schools to ensure that parents understand the importance of seeking specialized TBI care to prevent long-term consequences.

## Figures and Tables

**Table 1:** Sample demographics and injury characteristics.

Characteristic	Mean (*SD*)	*n* (%)
Child age (years)	3.60 (1.22)	
Child gender (% female)		20 (55.6%)
Child race		
White		11 (73.3%)
African American		4 (26.7%)
Child ethnicity (% Hispanic)		13 (86.7%)
Mechanism of Injury		
Fall		28 (77.8%)
MVC		2 (5.6%)
Direct hit		6 (16.7%)
Loss of consciousness		9 (25%)
Relationship to child (% mothers)		36 (100%)
Parent age (years)	29.47 (5.37)	
Parent race		
White		12 (80%)
African American		3 (20%)
Parent ethnicity (% Hispanic)		11 (73.3%)
Parent marital status		
Never married		7 (46.7%)
Separated		2 (13.3%)
Divorced		1 (6.7%)
Married		5 (33.3%)
Parent highest education obtained		
Some high school		1 (6.7%)
High school graduate		1 (6.7%)
Some college/Associate degree		8 (53.3%)
Bachelor’s degree		3 (20%)
Graduate degree		2 (13.3%)
Post-concussive symptoms		
Behavior		16 (44.4%)
Nausea/Vomiting		15 (41.7%)
Headache		14 (38.9%)
Balance		9 (25%)
Drowsiness		8 (22.2%)
Sadness		7 (19.4%)
Difficulty falling asleep		5 (13.9%)
Dizziness		4 (11.1%)
Slowed down		4 (11.1%)
Sensitivity to noise		4 (11.1%)
Sleeping less		4 (11.1%)
Irritability/Difficult to console		3 (8.3%)
Sleeping more		2 (5.6%)
Visual		2 (5.6%)
Sensitivity to light		2 (5.6%)
Memory issues		2 (5.6%)
Sought follow-up care		16 (44.4%)
Followed up with a TBI specialist		5 (33.3%)

Note. Injury characteristics, post-concussive symptoms, and follow-up data were available for the full sample included in the present study (*n* = 36). Full demographic data were available only for participants who enrolled in the larger PCIT study and completed the baseline visit (*n* = 15); the remaining 21 participants completed screening but did not enroll in the larger study.

**Table 2:** Multiple regression predicting medical follow-up.

Predictor	*B*	*SE B*	*β*	*t*	*p*
Headache	0.185	0.161	0.181	1.146	0.261
Drowsiness	0.414	0.190	0.347	2.177	* 0.037
Balance	0.456	0.162	0.397	2.821	* 0.008
Slowed down	−0.058	0.215	−0.046	−0.269	0.790
Behavior	0.181	0.157	0.181	1.154	0.258

Note. *n* = 36. The dependent variable was whether the child received medical follow-up. *R*^2^ = 0.504, adjusted *R*^2^ = 0.422, *F*(5,30) = 6.11, *p* = 0.001. *B* = unstandardized regression coefficient; *SE B* = standard error of the unstandardized coefficient; *β* = standardized regression coefficient. * *p* < 0.05.

## Data Availability

The data presented in this study are available on request from the corresponding author, the data are not publicly available due to privacy or ethical restrictions.

## References

[1] Faul M.D., Wald M.M., Xu L., Coronado V.G. (2010). Traumatic Brain Injury in the United States: Emergency Department Visits, Hospitalizations, and Deaths, 2002–2006.

[2] Centers for Disease Control and Prevention (2019). Surveillance Report of Traumatic Brain Injury-Related Emergency Department Visits, Hospitalizations, and Deaths—United States, 2014.

[3] Collins C.L., Yeates K.O., Pommering T.L., Andridge R., Coronado V.G., Gilchrist J., Comstock R.D. (2014). Direct medical charges of pediatric traumatic brain injury in multiple clinical settings. Inj. Epidemiol..

[4] Haarbauer-Krupa J., Lee A.H., Bitsko R.H., Zhang X., Kresnow-Sedacca M. (2018). Prevalence of Parent-Reported Traumatic Brain Injury in Children and Associated Health Conditions. JAMA Pediatr..

[5] Anderson V., Godfrey C., Rosenfeld J.V., Catroppa C. (2012). 10 years outcome from childhood traumatic brain injury. Int. J. Dev. Neurosci..

[6] Yeates K.O., Tang K., Zhang C., Beauchamp M.H., Craig W.R., Doan Q., Freedman S.B., Gravel J., Ware A.L., Zemek R.L. (2025). Pediatric Emergency Research Canada (PERC) A-CAP Study Team. Symptom Trajectories and Their Biopsychosocial Correlates in Pediatric Concussion: An A-CAP Study. J. Neurotrauma.

[7] Chandler M.C., Bloom J., Fonseca J., Ramsey K., De Maio V.J., Callahan C.E., Register-Mihalik J.K. (2023). Quality of Life Differences in Children and Adolescents With 0, 1 to 2, or 3+ Persistent Postconcussion Symptoms. J. Athl. Train..

[8] Novak Z., Aglipay M., Barrowman N., Yeates K.O., Beauchamp M.H., Gravel J., Freedman S.B., Gagnon I., Gioia G., Boutis K. (2016). Association of Persistent Postconcussion Symptoms With Pediatric Quality of Life. JAMA Pediatr..

[9] Bressan S., Takagi M., Anderson V., Davis G.A., Oakley E., Dunne K., Clarke C., Doyle M., Hearps S., Ignjatovic V. (2016). Protocol for a prospective, longitudinal, cohort study of postconcussive symptoms in children: The Take CA Re (Concussion Assessment and Recovery Research) study. BMJ Open.

[10] Jones K.M., Ameratunga S., Starkey N.J., Theadom A., Barker-Collo S., Ikeda T., Feigin V.L. (2021). Psychosocial functioning at 4-years after pediatric mild traumatic brain injury. Brain Inj..

[11] Riemann L., Voormolen D.C., Rauen K., Zweckberger K., Unterberg A., Younsi A. (2021). Persistent postconcussive symptoms in children and adolescents with mild traumatic brain injury receiving initial head computed tomography. J. Neurosurg. Pediatr..

[12] Slomine B.S., McCarthy M.L., Ding R., MacKenzie E.J., Jaffe K.M., Aitken M.E., Durbin D.R., Christensen J.R., Dorsch A.M., Paidas C.N. (2006). Health care utilization and needs after pediatric traumatic brain injury. Pediatrics.

[13] McCrory P., Meeuwisse W., Dvorak J., Aubry M., Bailes J., Broglio S., Cantu R.C., Cassidy D., Echemendia R.J., Castellani R.J. (2017). Consensus statement on concussion in sport—The 5th international conference on concussion in sport held in Berlin, October 2016. Br. J. Sports Med..

[14] Meehan W.P., Mannix R. (2010). Pediatric concussions in United States emergency departments in the years 2002 to 2006. J. Pediatr..

[15] Grubenhoff J.A., Deakyne S.J., Comstock R.D., Kirkwood M.W., Bajaj L. (2015). Outpatient follow-up and return to school after emergency department evaluation among children with persistent post-concussion symptoms. Brain Inj..

[16] Jimenez N., Ebel B.E., Wang J., Koepsell T.D., Jaffe K.M., Dorsch A., Durbin D., Vavilala M.S., Temkin N., Rivara F.P. (2013). Disparities in disability after traumatic brain injury among Hispanic children and adolescents. Pediatrics.

[17] Kooper C.C., van Houten M.A., Niele N., Aarnoudse-Moens C., van Roermund M., Oosterlaan J., Plötz F.B., Königs M. (2024). Long-Term Neurodevelopmental Outcome of Children With Mild Traumatic Brain Injury. Pediatr. Neurol..

[18] Meagher A.D., Beadles C.A., Doorey J., Charles A.G. (2015). Racial and ethnic disparities in discharge to rehabilitation following traumatic brain injury. J. Neurosurg..

[19] Garcia D., Rodríguez G.M., Lorenzo N.E., Coto J., Blizzard A., Farias A., Smith N.D.W., Kuluz J., Bagner D.M. (2021). Intensive Parent–Child Interaction Therapy for children with traumatic brain injury: Feasibility study. J. Pediatr. Psychol..

[20] Sady M.D., Vaughan C.G., Gioia G.A. (2014). Psychometric characteristics of the postconcussion symptom inventory in children and adolescents. Arch. Clin. Neuropsychol..

[21] National Center for Injury Prevention and Control (2003). Report to Congress on Mild Traumatic Brain Injury in the United States: Steps to Prevent a Serious Public Health Problem.

[22] McCrea M., Guskiewicz K., Randolph C., Barr W.B., Hammeke T.A., Marshall S.W., Powell M.R., Ahn K.W., Wang Y., Kelly J.P. (2013). Incidence, clinical course, and predictors of prolonged recovery time following sport-related concussion in high school and college athletes. J. Int. Neuropsychol. Soc..

[23] Yeates K.O., Kaizar E., Rusin J., Bangert B., Dietrich A., Nuss K., Wright M., Taylor H.G. (2012). Reliable change in postconcussive symptoms and its functional consequences among children with mild traumatic brain injury. Arch. Pediatr. Adolesc. Med..

[24] Taylor H.G., Dietrich A., Nuss K., Wright M., Rusin J., Bangert B., Minich N., Yeates K.O. (2010). Post-concussive symptoms in children with mild traumatic brain injury. Neuropsychology.

[25] Fridman L., Scolnik M., Macpherson A., Rothman L., Guttmann A., Grool A.M., Duque D.R., Zemek R.L. (2018). Annual trends in follow-up visits for pediatric concussion in emergency departments and physicians’ offices. J. Pediatr..

[26] Lumba-Brown A., Yeates K.O., Sarmiento K., Breiding M.J., Haegerich T.M., Gioia G.A., Turner M., Benzel E.C., Suskauer S.J., Giza C.C. (2018). Diagnosis and management of mild traumatic brain injury in children: A systematic review. JAMA Pediatr..

[27] Wetjen N.M., Pichelmann M.A., Atkinson J.L. (2010). Second impact syndrome: Concussion and second injury brain complications. J. Am. Coll. Surg..

[28] Stevens P.K., Penprase B., Kepros J.P., Dunneback J. (2010). Parental recognition of postconcussive symptoms in children. J. Trauma Nurs..

[29] Dupont D., Tang K., Beaudoin C., Dégeilh F., Gagnon I., Yeates K.O., Rose S.C., Gravel J., Burstein B., Stang A.S. (2024). Postconcussive symptoms after early childhood concussion. JAMA Netw. Open.

[30] Yengo-Kahn A.M., Wallace J., Jimenez V., Totten D.J., Bonfield C.M., Zuckerman S.L. (2021). Exploring the outcomes and experiences of Black and White athletes following a sport-related concussion: A retrospective cohort study. J. Neurosurg. Pediatr..

[31] Zonfrillo M.R., Master C.L., Grady M.F., Winston F.K., Callahan J.M., Arbogast K.B. (2012). Pediatric providers’ self-reported knowledge, practices, and attitudes about concussion. Pediatrics.

[32] Karver C.L., Wade S.L., Cassedy A., Taylor H.G., Stancin T., Yeates K.O., Walz N.C. (2012). Age at injury and long-term behavior problems after traumatic brain injury in young children. Rehabil. Psychol..

